# Applying 3D-printed prostheses to reconstruct critical-sized bone defects of tibial diaphysis (> 10 cm) caused by osteomyelitis and aseptic non-union

**DOI:** 10.1186/s13018-024-04926-2

**Published:** 2024-07-20

**Authors:** Bingchuan Liu, Liwei Wang, Xingcai Li, Zhuo Chen, Guojin Hou, Fang Zhou, Caimei Wang, Yun Tian

**Affiliations:** 1 Department of Orthopaedics, Peking University Third Hospital, Beijing, China; 2 Engineering Research Center of Bone and Joint Precision Medicine, Ministry of Education, Beijing, China; 3 Department of Anesthesiology, Peking University Third Hospital, 49 North Garden Rd, Haidian District, Beijing, 100191 China; 4 Beijing AKEC Medical Co., Ltd, Beijing, China

**Keywords:** Tibial critical-sized defect, 3D printing technology, Osteomyelitis, Aseptic non-union

## Abstract

**Background:**

Clinical repair of critical-sized bone defects (CBDs) in the tibial diaphysis presents numerous challenges, including inadequate soft tissue coverage, limited blood supply, high load-bearing demands, and potential deformities. This study aimed to investigate the clinical feasibility and efficacy of employing 3D-printed prostheses for repairing CBDs exceeding 10 cm in the tibial diaphysis.

**Methods:**

This retrospective study included 14 patients (11 males and 3 females) with an average age of 46.0 years. The etiologies of CBDs comprised chronic osteomyelitis (10 cases) and aseptic non-union (4 cases), with an average defect length of 16.9 cm. All patients underwent a two-stage surgical approach: (1) debridement, osteotomy, and cement spacer implantation; and (2) insertion of 3D-printed prostheses. The interval between the two stages ranged from 8 to 12 weeks, during which the 3D-printed prostheses and induced membranes were meticulously prepared. Subsequent to surgery, patients engaged in weight-bearing and functional exercises under specialized supervision. Follow-up assessments, including gross observation, imaging examinations, and administration of the Lower Extremity Functional Scale (LEFS), were conducted at 3, 6, and 12 months postoperatively, followed by annual evaluations thereafter.

**Results:**

The mean postoperative follow-up duration was 28.4 months, with an average waiting period between prosthesis implantation and weight-bearing of 10.4 days. At the latest follow-up, all patients demonstrated autonomous ambulation without assistance, and their LEFS scores exhibited a significant improvement compared to preoperative values (30.7 vs. 53.1, *P* < 0.001). Imaging assessments revealed progressive bone regeneration at the defect site, with new bone formation extending along the prosthesis. Complications included interlocking screw breakage in two patients, interlocking screw loosening in one patient, and nail breakage in another.

**Conclusions:**

Utilization of 3D-printed prostheses facilitates prompt restoration of CBDs in the tibial diaphysis, enabling early initiation of weight-bearing activities and recovery of ambulatory function. This efficacious surgical approach holds promise for practical application.

**Supplementary Information:**

The online version contains supplementary material available at 10.1186/s13018-024-04926-2.

## Introduction

Tibial fractures constitute a significant portion, approximately 37%, of all lower limb fractures and occur at a rate of 17–21% per 100,000 individuals [1, 2]. Due to factors such as inadequate soft tissue coverage, poor blood supply, and even open trauma, the tibial diaphysis is predisposed to non-union and osteomyelitis following fracture, potentially leading to the development of critical-sized bone defects (CBDs) [2]. Tibial CBDs refer to defects that cannot heal without additional intervention. If left untreated, these defects can result in leg-length disparities, deformities, joint stiffness, and functional impairment, and have substantial adverse effects on the patients’ quality of life, sometimes necessitating limb amputation due to illness, psychological stress, and financial strain [3, 4].

Although bone grafting, including autogenous, allogeneic, and artificial bone material grafting, has long been considered the “gold standard” for bone defect repair [5–7], its applicability is more suitable for small defects with well-preserved soft tissue envelopes [8]. Moreover, limitations such as limited supply, donor-site morbidity, disease transmission, and foreign body rejection persist. When a defect reaches the “critical size” threshold, simple bone grafting may prove ineffective.

In recent decades, the Masquelet technique and Ilizarov bone transport have emerged as viable options for treating tibial CBDs [9–11]. The Masquelet technique involves a two-stage process of debridement with cement spacer insertion followed by bone grafting. Ilizarov bone transport entails debridement and shortening of the affected bone, followed by progressive lengthening using an external fixation frame. However, the therapeutic effectiveness of these methods can be compromised by complications such as pin tract and donor site infections, non-union, mechanical issues, and even refractures [12–14]. Many patients are unable to engage in weight-bearing exercises during the early postoperative period. A recent meta-analysis revealed that the final union time in the Masquelet and Ilizarov groups averaged at 7.59 and 12.22 months, respectively, while the time to full weight bearing averaged at 7.67 and 9.45 months, respectively [15].

3D-printed prosthesis implantation is a newly-developed method for reconstructing the tibial CBDs [8, 16]. This technique enables rapid restoration of anatomical integrity and biological stress conduction of the tibia, allowing for early weight-bearing after surgery. Several studies have reported favorable clinical outcomes following the implantation of 3D-printed prostheses to repair tibial CBDs resulting from comminuted fractures [17], non-union [18], osteomyelitis [19], and tumor resection [20]. However, these studies primarily consist of isolated case reports lacking comprehensive introductions to the associated surgical effects and complications. To date, no systematic research has specifically investigated the treatment outcomes of utilizing 3D-printed prostheses to repair CBDs in the tibial diaphysis.

This study aims to investigate the clinical feasibility and effectiveness of employing 3D-printed prostheses to repair CBDs in the tibial diaphysis exceeding 10 cm in length. To our knowledge, this is the first study reported specifically on the repair of tibial diaphyseal CBDs with 3D-printed prostheses. The results will help to systematically clarify the surgical experience, clinical outcomes and complications, providing meaningful references for clinical decision-making and surgical practice.

## Materials and methods

### Patient enrollment

This study was ethically approved by the Medical Science Research Ethics Committee of our institution (No. M2018174) and registered at ClinicalTrials.gov PRS (No. NCT03941028). Informed consents were obtained from all patients and their family members.

The inclusion criteria were as follows: (1) CBDs resulting from aseptic non-union or osteomyelitis; (2) CBD length > 10 cm; and (3) age > 18 years. Exclusion criteria were as follows: (1) tumor-related CBDs, (2) CBDs located at the tibial metaphysis or invading the articular surface, and (3) inability to complete the entire treatment and follow-up process. Detailed patient data, including age, sex, cause and length of defects, pathogenic bacteria, comorbidities, imaging examinations, limb functions, and complications, were retrospectively collected.

### Surgical protocol

**Fig. 1:**
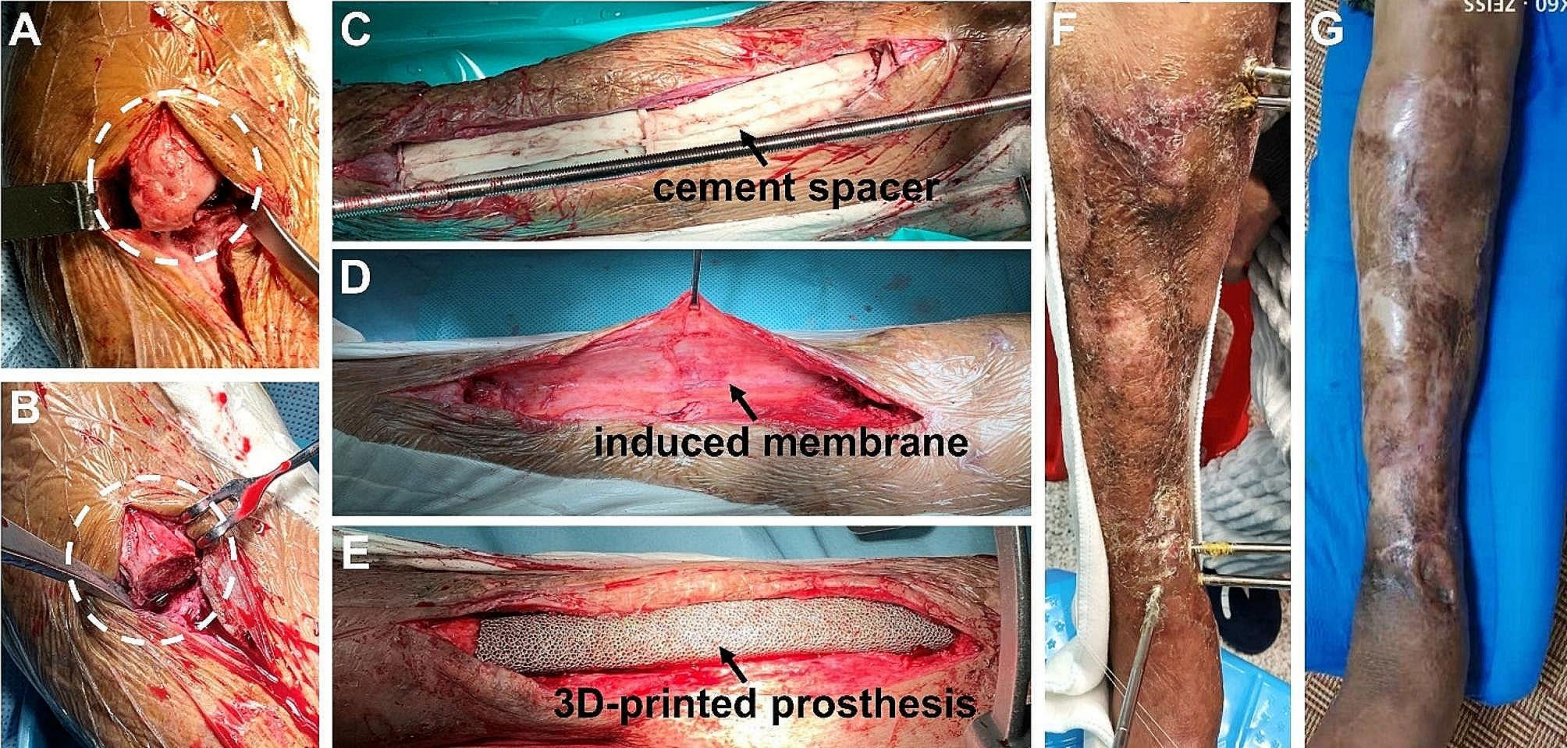
Surgical steps and gross observation: (**A**) necrotic bone hyperplasia at the defect end; (**B**) cut the hyperplastic bone; (**C**) bone defect was filled with cement spacer; (**D**) after removal of the cement spacer, the surrounding induced membrane could be observed; (**E**) the 3D-printed prosthesis was inserted inside the tibial bone defect; (**F**) a preoperative gross photo showed obvious skin contracture and varus deformity of the crus; (**G**) a postoperative gross photo showed that the shortening and varus deformity significantly improved

**Debridement and spacer implantation**. Initially, necrotic or infected soft tissue was thoroughly excised. The infected bones and sequestra were then debrided until cortical bleeding (paprika sign) was achieved (Fig. 1A and B). Subsequently, the tibia was stabilized with an external frame in a neutral position, and a pre-shaped polymethyl methacrylate (PMMA) cement spacer was inserted to fill the defect space (Fig. 1C). The cement powder was mixed with vancomycin in a ratio of 20:1. Vacuum-sealed drainage (VSD) was employed for infected lesions, facilitating continuous irrigation and aspiration within the bone defect areas and soft tissue layers. Once infection resolution was confirmed, wounds were closed, and skin grafting or flap transfer was performed if necessary. For infected CBDs, repeated debridement and cement spacer replacement were conducted weekly until infection resolution.

**Fig. 2:**
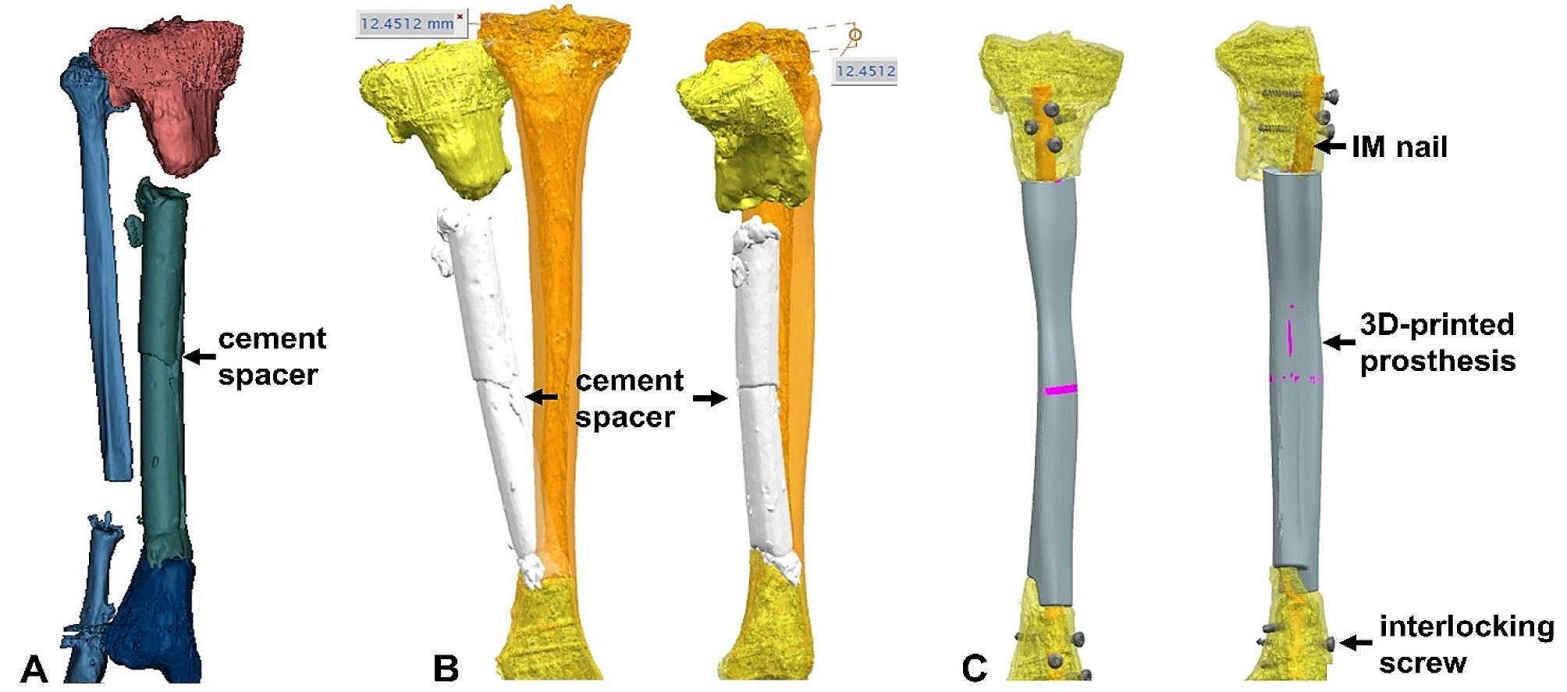
The design process of a 3D-printed prosthesis: (**A**) 3D modeling of the affected limb; (**B**) according to the principle of mirror symmetry, the size and shape of a prosthesis was designed guided by the healthy tibia (the orange tibia represented the healthy side); (**C**) 3D simulation of prosthesis implantation and internal fixation

**Prosthesis design and fabrication**. External contour design of the prosthesis was based on the mirror contrast principle, with radiography and computed tomography (CT) scans of bilateral tibiae guiding the design process. The internal space of the prosthesis featured interconnected porosity with a pore size of 625 ± 70 μm and porosity of 68%. Fabrication was carried out via selective laser melting (Arcam EBM, Gothenburg, Sweden) using standard Ti6Al4V powders. The elasticity modulus of the prosthesis was 1200 ± 48 MPa, close to trabecular bone and significantly lower than cortical bone, which is highly advantageous to eliminate the stress shielding (Fig. 2).

**Prosthesis implantation and internal fixation**. Following confirmation of no recurrence of infection, the prepared 3D-printed prosthesis was implanted after 6–8 weeks. During incision, the induced membrane was protected to enhance prosthesis stability and bone regeneration in subsequent stages (Fig. 1D). The prosthesis was implanted inside the bone defect and fixed by intramedullary (IM) nails and interlocking screws (Fig. 1E). For patients with defects > 20 cm in length or poor bone quality, cortical screws were utilized to reinforce the prosthesis flank. No additional bone grafting was performed inside or around the prosthesis.

Scanning electron microscope (SEM) was used to observe the microstructure of the prosthesis surface. As shown in Supplementary Fig. 1A, we could observe that the pores of the prosthesis were uniform in size and distribution, and no obvious powders and debris existed. Supplementary Fig. 1A and 1B displayed the gross types of the prosthesis, nail and screws. The basic materials of prostheses, nails, screws were all titanium alloy. Their elasticity modulus and Poisson’s ratio of titanium alloy were 110 GPa and 0.34.

### Rehabilitation and follow-up

Following surgery, patients underwent functional rehabilitation under professional guidance. Gradual weight-bearing was initiated from the first few days, with moderate limb weight bearing permitted using a walker or cane. Full weight-bearing was gradually progressed based on radiological assessments. Weight-bearing was appropriately postponed in patients with poor bone quality or age-related osteoporosis. Patients were followed up at 3, 6, and 12 months within the first year, and annually thereafter. Follow-up included gross observation, imaging examinations, and quantitative evaluation of limb function using the Lower Extremity Functional Scale (LEFS).

### Statistical analysis

Statistical analysis was performed using SPSS version 22.0. Variable data are presented as means ± standard deviation and were assessed using the Kruskal–Wallis test. Attribute data are presented as numbers (percentages) and were assessed using t-tests. Statistical significance was set at *P*-value < 0.05.

## Results

The clinical data of the included cases are listed in Table 1. Fourteen patients with tibial CBDs (11 men and 3 women) were enrolled in this study, with an average age of 46.0 ± 12.1 years (range: 34–65 years). Four patients had comorbidities, including hypertension, diabetes, and gout. The causes of CBDs comprised chronic osteomyelitis (10 patients) and aseptic non-union (4 patients). The average length of defects was 16.9 ± 4.8 cm (range: 10.6–22.9). Three patients underwent Ilizarov bone transport before 3D-printed prosthesis implantation, which proved unsuccessful. The average time interval between prosthesis implantation and limb weight bearing for these 13 patients was 10.6 ± 4.0 days (range: 6–22). The average follow-up period for all patients was 28.4 ± 11.9 months (range: 16–56). In terms of functional rehabilitation, all patients could walk autonomously without assistance at the last follow-up, and their Lower Extremity Functional Scale (LEFS) scores at the last follow-up were significantly higher than those before surgery (30.7 vs. 53.1, *P* < 0.001).

**Table 1:** Summary of included cases

	Gender/Age (years)	Cause	Pathogenic bacteria	Comorbidities	Defect length (cm)	Time to limb weight-bearing (days)	Surgery-related complications	LEFS		F/U (months)
								pre-op	last F/U	
1	M/65	COM	enterobacter cloacae, escherichia coli	hypertension, diabetes	10.6	13	broken screws	32	62	56
2	M/32	COM	staphylococcus aureus	none	12.4	10	broken screws	22	48	50
3	M/65	aseptic nonunion	none	none	22.9	14	broken IM nail	23	44	40
4	F/34	COM	staphylococcus aureus, pyogenic streptococcus	none	21.8	13	none	43	57	33
5	F/35	aseptic nonunion	none	Post Ilizarov technique	11.5	6	none	32	52	28
6	M/38	COM	klebsiella aerogenes	Post Ilizarov technique	20.5	7	none	42	58	28
7	M/53	aseptic nonunion	none	diabetes, gout	11.8	7	none	36	55	25
8	M/62	COM	staphylococcus aureus	hypertension	12.5	12	none	33	54	23
9	F/34	COM	enterococcus durans	none	20.6	7	loose screw	40	55	22
10	M/51	COM	enterobacter cloacae	Diabetes Post Ilizarov technique	22.2	7	none	22	52	21
11	M/47	COM	pseudomonas aeruginosa, staphylococcus aureus	none	12.4	10	none	26	50	20
12	M/34	COM	staphylococcus aureus	none	15.1	10	none	35	55	20
13	M/57	aseptic nonunion	none	none	21.5	10	none	24	54	16
14	M/37	COM	klebsiella aerogenes, escherichia coli	none	21.9	22	none	20	48	16

**Notes**: LEFS: lower extremity functional scale; F/U: follow-up; M: male; F: female; COM: chronic osteomyelitis; pre-op: preoperative; IM nail: intramedullary nail

**Fig. 3:**
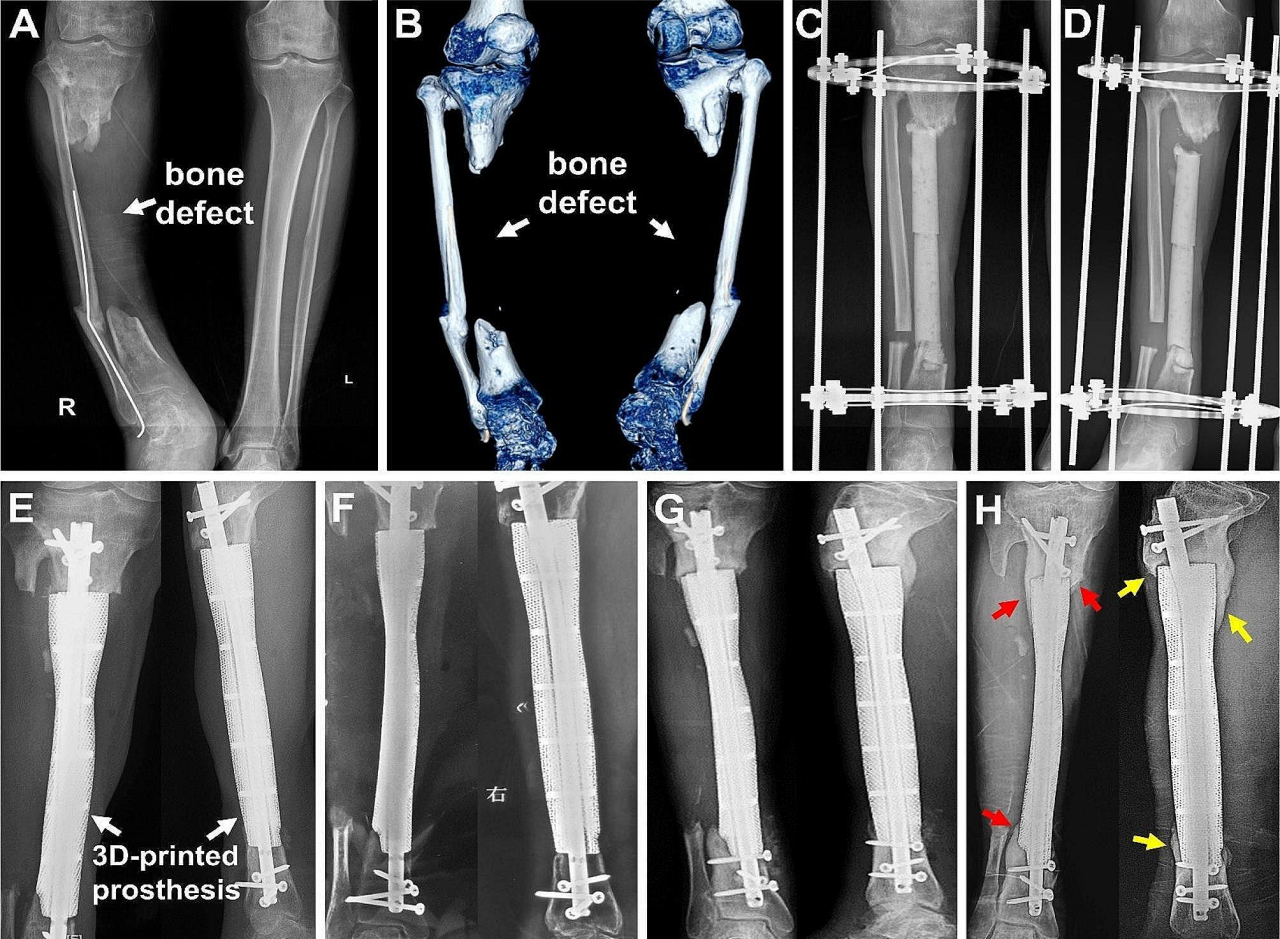
**A** typical case: (**A**) a preoperative X-ray showed the critical-sized defect of right tibia (22.9 cm), accompanied with tibial shortening and varus deformity; (**B**) preoperative 3D reconstruction CT images further demonstrated the characteristics of tibial defect and deformity; (**C**) after the debridement and osteotomy, the defect was filled with a cement spacer, and a circular external frame was used to stabilize and correct the tibial deformity; (**D**) after 2 months of continuous traction, the tibia was lengthened by 2 cm; (E-H) a 3D-printed prosthesis was inserted inside to reconstruct the tibial critical-sized defect, as shown by the continuous anteroposterior and lateral X-ray follow-ups, at 2 weeks (**E**), 3 months (**F**), 15 months (**G**) and 38 months (**H**) postoperatively, new bone gradually grew along the prosthesis, and no looseness or breakage occurred (red and yellow arrows represent the new bone callus in the anteroposterior and lateral X-rays)

Four patients experienced surgery-related complications, including broken nails (one case), broken interlocking screws (two cases), and loose interlocking screws (one case). No prosthesis breakage or subsidence occurred. The broken nail was detected after impact trauma, leading to reoperation to replace the intramedullary (IM) nail. The other three complications were identified during routine follow-up procedures. Regarding bone regeneration, new calluses gradually developed from the defect end towards the central area (Fig. 3). The associated imaging characteristics included the following observations: (1) initial proliferation of new calluses at the defect end, forming a pedestal-like bone structure and establishing local stability; (2) continued crawling of new bone along the prosthesis surface, albeit at a gradually reduced growth rate, resulting in a thinning of the new bone thickness; and (3) observation of low-density transparent lines between the newly formed bone and the prosthesis, making it challenging to determine whether new bone had grown within the porous structure of the prosthesis.

## Discussion

In this retrospective study, we present the surgical experience and clinical outcomes of utilizing 3D-printed prostheses for repairing tibial diaphyseal CBDs. Our findings highlight the advantages of this technology while identifying surgery-related complications that warrant further optimization.

Applying 3D printing technology to clinical practice requires a concerted effort from both clinicians and engineers to accomplish the complex process of design, manufacturing, and the layer-by-layer construction of customized models [21, 22]. In the early stages of utilizing 3D printing to enhance surgical procedures, clinicians employed 3D-printed polymer models to clearly delineate the lesion morphology and the adjacent anatomical structures, facilitating more accurate surgical planning and execution [23, 24]. With advancements in metal-related 3D printing technology, the innovative use of 3D-printed metal prostheses to repair bone defects has been increasingly adopted in clinical settings, including defects in the mandible, sternum, vertebrae, and limbs [17–19, 25–27]. 3D-printed metal prostheses not only enable precise repair of the anatomical structure of bone defects but also provide sufficient mechanical strength to restore the biomechanical properties, mobility, and load-bearing function of bones.

Aseptic and septic non-union represent the primary causes of CBDs in the tibial diaphysis. The tibia bears the highest risk of non-union among long bones, with an annual incidence ranging from 12 to 19% [28]. Managing septic non-union, especially in the presence of chronic osteomyelitis and bacterial biofilms, poses significant challenges, as conventional antibiotics may be ineffective [29]. We determined that effective infection control is crucial for prosthesis implantation. We used extended osteotomy, VSD drainage, and an antibiotic-cement spacer to achieve effective infection control. Full osteotomy completely cleared the infected lesions and biofilms and restored blood supply to the bone defect area. VSD can improve local blood circulation, promote the growth of granulation tissue and wound healing, reduce the exudation of tissue fluids, and maintain wound moisture [30].

All 14 enrolled patients engaged in limb weight-bearing exercises early postoperatively, with an average interval of 10.6 days. These patients experienced significant limb function recovery, with their LEFS scores at the last follow-up significantly higher than those pre-surgery (*P* < 0.001). This improvement can be largely attributed to the anatomically matched 3D-printed prostheses with sufficient biomechanical strength. The modulus of elasticity of our prostheses closely resembles trabecular bone and is significantly lower than cortical bone, facilitating stress conduction while avoiding stress shielding. Unlike traditional treatments where weight-bearing rehabilitation is delayed until bone healing, 3D-printed prostheses enable stable stress conduction without relying on bone healing, allowing for safe weight-bearing. Additionally, employing medial IM nail fixation aids in central axial stress conduction, minimizing prosthesis eccentric displacement and tibial varus deformity [31, 32].

New bone regeneration after prosthesis implantation is closely related to the long-term stability of the prosthesis and tibial stress conduction. According to our radiological F/Us, the new bone could proliferate at the defect and crawl on the prosthesis surface; however, the growth rate and new bone thickness gradually decreased. The proliferation of new bone is related to the local micromovement between the prostheses and bone planes. Less rigid fixation enhances fracture healing; the optimal interfragmentary movement at the fracture site is 0.2–1.0 mm [33]. The elastic fixation mode provided by the IM nails allowed for micro-movement, promoting a larger amount of local bone proliferation.

The progression of bone regeneration is associated with several factors. An abundant blood supply can provide the necessary nutrients for new bone regeneration and metabolic waste transport. However, because of poor soft tissue coverage and repeated debridement, tibial CBDs are often associated with poor local blood supply. All 14 patients in our study had soft tissue contractures, and eight underwent flap transplantation because of large skin defects. To mitigate the negative effects of poor soft tissue coverage, Masquelet’s technique was applied during the surgical process in which PMMA cement spacers were implanted inside the defect areas to induce the membrane. According to previous studies, the induced membrane is composed of a fibroblast/collagen matrix with an inner synovial-like epithelium and a vascularised outer layer and is rich in mesenchymal stem cells, leucocytes, and osteoclasts [34–36]. After the prosthesis was implanted, the induced membrane could gradually secrete osteoinductive factors, such as BMP-2, and growth factors, such as VEGF and TGF-β1, to promote revascularisation and bone regeneration. According to Wolff’s law, bone regeneration and remodelling occur due to mechanical stress [37, 38]. Therefore, we encouraged patients to perform weight-bearing exercise early after surgery to increase the micromotion and axial stress between the prosthesis and bone, thereby simulating new bone regeneration.

New bone growth inside the porous structure of 3D-printed prostheses to form osseointegration affects medium- and long-term outcomes [39]. In our study, the overlapping effect of two-dimensional radiographs and mental artifacts from the prosthesis resulted in the observation of low-density translucent lines between the new bone and the prosthesis. This phenomenon made it challenging to conclusively determine whether the new bone had indeed grown inside the porous structure of the prosthesis. Histopathological staining is typically regarded as the gold standard for confirming osseointegration [40, 41]. However, due to not having the prosthesis been removed, we were unable to obtain suitable clinical specimens for histopathological staining. These aspects will be thoroughly analyzed and reported in future studies.

Common postoperative complications in prosthesis-implantation surgeries include infection and aseptic loosening [42, 43], neither of which occurred in our case series. In this study, four patients experienced surgery-related complications. One case of IM nail breakage was caused by an unfortunate impact trauma and underwent reoperation, one case of screw loosening was related to poor bone quality and insufficient screw-holding force, and two cases of interlocking screw breakage may have been related to the axial instability of the prosthesis and excessive stress bearing. Based on these complications, we added lateral flank fixation strength in patients with poor bone quality and axial stability and appropriately slowed down the starting time of weight bearing. No postoperative infection recurrence was observed in our patient series. The percentages of infection recurrence were 1.6%, 14.4%, and 7.0% in the Ilizarov bone transport, Masquelet, and vascularised fibular graft groups, respectively [44]. Our technical advantage was the personalised design and manufacture of the 3D-printed prostheses, which ensured a wider range of infected bone removal and sequestrum osteotomy during debridement, thereby reducing infection recurrence.

Our study has some limitations. First, the number of patients included was relatively small, and a longer F/U time was required to evaluate the long-term clinical outcomes of using 3D-printed prostheses to repair tibial CBDs. This study did not include a control group. We plan to supplement and compare the treatment efficacy of different methods of repairing tibial CBDs in future studies.

## Conclusion

The application of 3D-printed prostheses proves to be an efficient method for reconstructing tibial diaphyseal CBDs associated with osteomyelitis and non-union. These prostheses aid in rapidly restoring tibial anatomical morphology and facilitating biological stress conduction. Over time, new bone gradually regenerates following prosthesis implantation. Patients can engage in early weight-bearing exercises post-implantation, leading to favorable limb function recovery. Nonetheless, the long-term clinical outcomes and bone regeneration patterns necessitate further observation. In summary, the implantation of 3D-printed prostheses emerges as an effective clinical option for repairing tibial diaphyseal CBDs.

### Electronic supplementary material

Below is the link to the electronic supplementary material.

Supplementary Material 1

## Data Availability

No datasets were generated or analysed during the current study.
