# Supplementary material for: Applying 3D-printed prostheses to reconstruct critical-sized bone defects of tibial diaphysis (> 10 cm) caused by osteomyelitis and aseptic non-union
Source: J Orthop Surg Res. 2024 Jul 20;19:418. doi: 10.1186/s13018-024-04926-2 (PMC11264997; doi:10.1186/s13018-024-04926-2)
Supplement: Supplementary file 1 — Supplementary Material 1 [file 13018_2024_4926_MOESM1_ESM.docx]

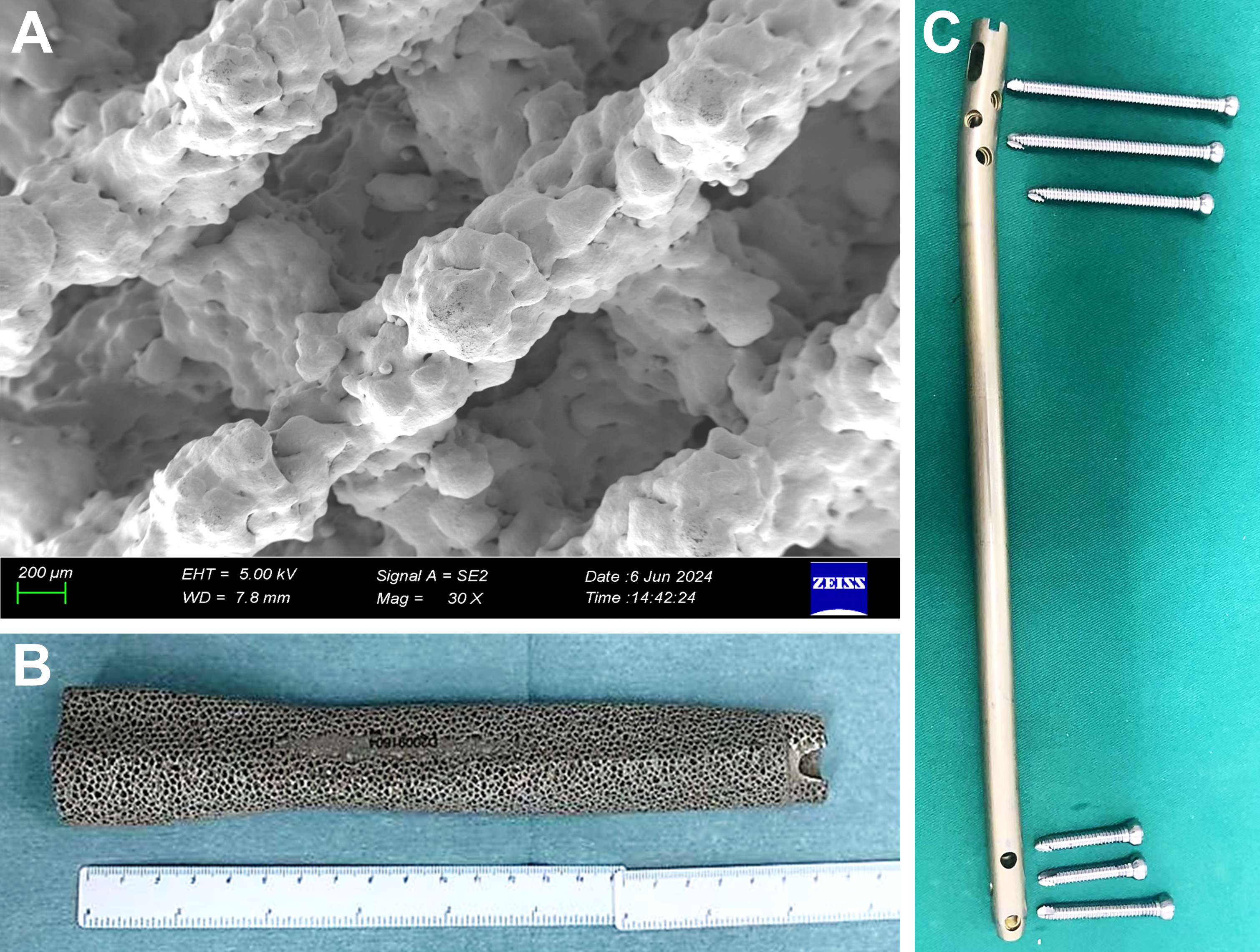


**Supplementary Figure 1**. Details of the prosthesis, nail and screws: (A) scanning electron microscope of the prosthesis surface; (B) gross view of the porous prosthesis; (C) gross view of the nail and interlocking screws.
